# Gelatin as It Is: History and Modernity

**DOI:** 10.3390/ijms24043583

**Published:** 2023-02-10

**Authors:** Oleg V. Mikhailov

**Affiliations:** Department of Analytical Chemistry, Certification and Quality Management, Kazan National Research Technological University, K. Marx Street 68, 420015 Kazan, Russia; ovm@kstu.ru

**Keywords:** protein, collagen, gelatin, biopolymer, synthesis, silver halide photographic process, immobilization, drug transport, nanoparticles

## Abstract

The data concerning the synthesis and physicochemical characteristics of one of the practically important proteins—gelatin, as well as the possibilities of its practical application, are systematized and discussed. When considering the latter, emphasis is placed on the use of gelatin in those areas of science and technology that are associated with the specifics of the spatial/molecular structure of this high-molecular compound, namely, as a binder for the silver halide photographic process, immobilized matrix systems with a nano-level organization of an immobilized substance, matrices for creating pharmaceutical/dosage forms and protein-based nanosystems. It was concluded that the use of this protein is promising in the future.

## 1. Introduction

Gelatin as a product of anthropogenic activity has been known since very ancient times: in any case, even then, it was noticed that meat and fish broths are sometimes able to spontaneously solidify in the air without any preliminary cooling with the formation of a specific substance, this was something in between a liquid and solid body. Already in the 10th century B.C. the book “Kitab al-Tabikh” described the recipe for the preparation of fish jelly, the main component of which is actually one of the varieties of gelatin, by boiling fish heads [1]. According to [2], the book “Viandier of Taillevent”, dated approximately 1375, presents the procedure for preparing a jellied meat broth, in which gelatin is also an integral part. In the 15th century, in Britain for the same purpose, meat production wastes and the hooves of cattle began to be used as raw material for the production of gelatin [3], and in 1681, the French inventor Denis Papin improved this technology by developing a new method for extracting gelatin by boiling bones [4]. Almost a century and a half later, another French researcher, namely Jean-Pierre-Joseph d’Arcet, 1812 continued the experiments of his predecessor by introducing hydrochloric acid in the process of extracting gelatin from bone raw materials in combination with steam extraction, which significantly increased its efficiency [5]. In this connection, it is interesting to note that even then, the French government considered gelatin as a potential source of cheap and affordable protein for the poorest strata of society [5], which significantly contributed to its production on an industrial scale. It is possible that this very French experience contributed to the fact that it was paid attention to in the USA, where gelatin gained particular popularity as a food product called Jell-O [6]. Along with this, since the middle of the 19th century, in this country, gelatin in powder form began to be produced and widely sold. This circumstance significantly expanded the possibilities of using this protein product [3]. At the present time, gelatin is perhaps the most demanded gelling agent in the food industry in general and cooking in particular, and its various types and varieties are used in the preparation of a wide range of food and non-food products. Examples of food products containing this protein are gelatin desserts, trifles, aspic, marshmallows, candy corn, and confections such as Peeps, gummy bears, fruit snacks, and jelly babies. Gelatin can be used as a stabilizer, thickener, or texturizer in products such as yoghurt, cream cheese, and margarine; it is also used in reduced-fat products to mimic the feeling of fat in the mouth and give the impression of bulk. It is also used in the production of several types of Chinese soup dumplings, specifically Shanghainese soup dumplings, or xiaolongbao, as well as Shengjian mantou, a type of fried and steamed dumplings.

Up to the 20th century, the use of gelatin as a food product remained, if not the only, then, in any case, the main direction of its practical use. This direction is undoubtedly one of the most important at the present time, too. However, it is only “the tip of the iceberg” because its capabilities in solving a wide variety of problems that arise within the framework of anthropogenic activity are much wider. These include such applications in the field of cultural activity as glue and adhesive compositions in painting, in the manufacture of handicraft and art products from wood, leather, textiles, books, newspapers, and magazines; as water-soluble shells for washing and dishwasher tablets, as a material for ballistic tests (so-called ballistic gelatin), etc. All these applications, however, are somehow related to the physical or mechanical characteristics of gelatin arrays, i.e., formed at the macro level. Those applications of gelatin that are associated with the characteristics of its constituent molecules and which are already formed at the micro- or nano-level of the organization of this substance are much less known. The present review will be devoted to the presentation of the data related namely to such applications. However, before presenting all these data, it is first necessary to carry out a detailed consideration of the technology for obtaining and the physicochemical characteristics of gelatin and at each of the three above levels of its organization—both at the macro- and at the micro-/nano-levels.

## 2. Specificity of Gelatin Synthesis

The foundations of the modern production of gelatin were laid mainly in the last 20th century. In general, the so-called connective tissue contained in skins and split skins, as well as the bones, tendons, and cartilage of cattle, is used as a raw material for the production of gelatin. The process of obtaining gelatin from such raw materials includes several stages, which are considered to some extent in a number of works (see, for example, [7,8,9,10,11,12]). Summarizing the data of these works, the technology for its production can be represented as follows.

*Primary processing*. The initial raw mass is necessarily preserved by adding ordinary table salt (sodium chloride) or slaked lime (calcium hydroxide); after that, it is re-preserved and crushed. Then, it must be treated with water or a solution of hydrochloric (hydrochloric) acid in order to remove the remnants of the preservative. The bulk of the fat contained in it is also removed from the raw material to a level of 1% (mass) and less, for which it is washed using hot water. Along with this, it is very important to remove from the raw mass the so-called “ballast” proteins (mucoids, albumins, globulins, etc.), which do not have the most favorable effect on the quality of the future target product (i.e., gelatin). For this purpose, one of the two operations is carried out:

- Treatment with acid or alkali solutions. During this processing, the “ballast” proteins contained in the raw material break down into smaller polypeptides and pass into the processing solution, and only the gelatin precursor, collagen, which does not undergo destruction (although it acquires a looser structure) remains in the processed mass.
- Treatment in a solution of calcium hydroxide (slaked lime). This variant is used for the skins of cattle and provides for the constant renewal of the solution of this reagent since the duration of the processing of raw materials with it is quite long (up to two months or even more). After the completion of this procedure, the above reagent is washed off, neutralized by the action of hydrochloric acid, and the resulting mass is again thoroughly washed with water.

*Hydrolysis*. Currently, any one of three collagen processing options is used at this stage: acidic, alkaline, and enzymatic. Acid treatment is carried out by influence on the collagen with sulfuric, hydrochloric, or phosphoric acid; it is used mainly on leather and skins, resulting in the so-called acid gelatin (referred to as type A). This hydrolysis variant is particularly well suited for materials such as porcine skin collagen and requires 10–48 h. Such gelatin is called type A. Alkaline processing is carried out by influencing the collagen to an aqueous solution of calcium hydroxide; it is used for more complex collagens, resulting in the so-called alkaline gelatin (type B). This treatment is most often used for collagen, which is found in bovine hides; however, it requires a longer duration (usually several weeks). The enzymatic hydrolysis of collagen to extract gelatin is a relatively new process. Its advantages are that the processing time is shorter than with alkaline or acid hydrolysis and also that gelatin degradation is minimized here. In addition, the gelatin obtained in this way has a higher purity compared to both acidic and alkaline gelatin.

*Extraction*. This stage is a multi-stage process, and the temperature of this process, as a rule, increases only at its final stages, which ensures the minimal thermal decomposition of the extracted gelatin. To realize it, special devices based on the fractional method are used; at the same time, the product obtained upon completion of the second of the above stages is poured with water at a temperature of (55–60) °C and extracted. After partial extraction, the process is repeated (with that part of the raw material that was extracted), but this time, water heated to a higher temperature is used. The whole technological process is repeated several more times until almost all the gelatin contained in it is extracted from the above product.

*Recovery*. This process includes filtration, evaporation, drying, grinding, and screening. First, the solutions obtained in the previous stage are thoroughly cleaned of all undesirable impurities present in them, as well as fatty inclusions. Separators and/or special filter presses are used as equipment for the purification of gelatin broths. The solutions that are purified in this way are thickened with the help of an ultrafiltration unit operating according to membrane filtration technology. As a result of this technological stage, the so-called “retentate”, which, in fact, is condensed gelatin, is obtained. (It should be noted in this connection that this produces another product, the so-called permeate, which is a water solution containing minerals and also a number of low molecular weight organic compounds). In the next stage, the concentrated gelatin solution is subjected to mandatory sterilization, for which the product is treated at a temperature of 130 °C. Then, the sterilized and maximally purified gelatin solutions are subjected to evaporation and subsequent solidification, as a result of which they take on a jelly-like form (jelly). The resulting jellies are then dried in a stream of purified air, crushed in special crushers, sieved, and thoroughly mixed until a homogeneous mass is obtained. To avoid degradation of the gelatin, a low temperature is used during the recovery process. Many recovery processes occur quickly, and all associated processes are carried out in stages to minimize the destruction of the gelatin structure since even its partial destruction leads to a decrease in the strength of the jelly (gel) formed by it.

In concluding this paragraph, it should be noted that, in recent years, various fish products (for example, shark cartilage) have become quite widely used in the production of gelatin as an alternative raw material since they eliminate some of the religious barriers associated with the production and consumption of gelatin [13,14,15].

## 3. Gelatin Physico-Chemistry

**Scheme 1:**
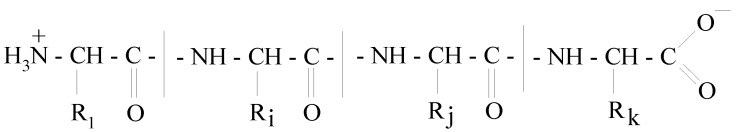
The structural formula of the gelatin.

Since, as mentioned above, gelatin is obtained from the natural fibrillar protein collagen, collagen is its precursor, which can be formally considered a kind of gelatin “anhydride”. Its hydrolytic conversion to gelatin produces molecules of various molecular weights, but each of them is a fragment of the collagen chain from which it was cleaved. Taking into account this circumstance, gelatin is not an individual chemical compound but a mixture of fractions consisting entirely of amino acids connected by peptide bonds to form low molecular weight polypeptides with the general formula (Scheme 1) (R_1_, R_i_, R_j_, R_k_ are various radicals) and molecular mass (*M*) from 15,000 and above [16,17,18,19] or their aggregates with *M* = 200.000–300.000 [20,21], which are composed of residues of 18 natural amino acids out of 20, with the exception of cystine and cysteine. Due to the high M values of the polypeptides that make up gelatin, it is often called a biopolymer, and we will also use this name from time to time in the future. [In fairness, however, it should be noted that the use of this term in relation to gelatin is not entirely correct. The point is that only those synthetic and natural high-molecular compounds fall under the concept of “polymer”, for which a regularly repeating structural unit can be indicated (such as for example, in polypropylene (–CH_2_–CH_2_–CH_2_–)_n_ or in natural rubber (–CH_2_–C(CH_3_)=CH_2_–)_n_), that does not take place in the case of gelatin]. Even more than 100 years ago, the quantitative composition of gelatin was established by means of gross chemical analysis [22]; according to the data of this work, in terms of the key chemical elements that make up its composition, it contains, on average (in wt.%): C—50.5, H—6.8, N—17.0 and O—25.2.

**Figure 1:**
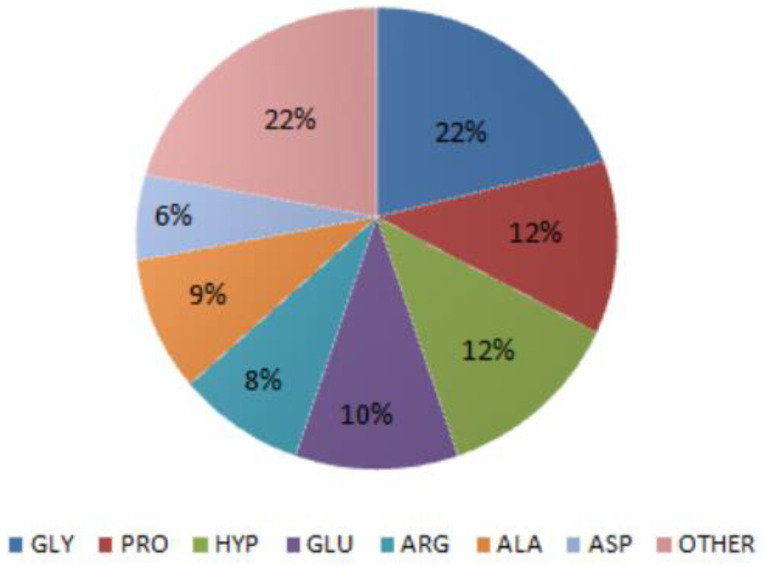
Average amino acid composition of gelatin (GLY—glycine, PRO—proline, HYP—hydroxyproline, GLU—glutamine, ARG—arginine, ALA—alanine, ASP—asparagine) (see website https://en.wikipedia.org/wiki/Gelatin) (Accessed on 10 December 2022).

Gelatin, similar to any protein, shows reactions that are typical of proteins and is hydrolyzed by most proteolytic enzymes to form a peptide or amino acid components [23]. Information on the amino acid composition of various varieties of gelatin was established already in the middle of the 20th century and can be found in a number of works, in particular [13,14,15,24,25,26,27,28,29,30], as well as in the previously mentioned book [12]. It should be noted immediately that the predominant part of these amino acid units of gelatin (slightly more than a third of the total) is the “residue” of the simplest amino acid glycine. A characteristic feature (as well as its “ancestor” collagen) that distinguishes it from other proteins is the unusually high content of the cyclic amino acid proline and hydroxyproline [31], which, according to this indicator, occupy the second and fourth positions, respectively (the third component with a noticeably lower content is alanine). The almost full absence among them of those amino acid fragments that contain sulfur (cystine and cysteine) clearly indicates that for the structure of both gelatin and collagen, groups with both the so-called labile sulfur (–SH) and disulfide bridges (–S–S–) are uncharacteristic. At the same time, non-polar or “hydrophobic” amino acid residues make up at least 2/3 of their total number, although gelatin, as is well known, is a hydrophilic substance. Some idea of the average amino acid composition of gelatin is given by the generalized diagram presented in Figure 1.

**Figure 2:**
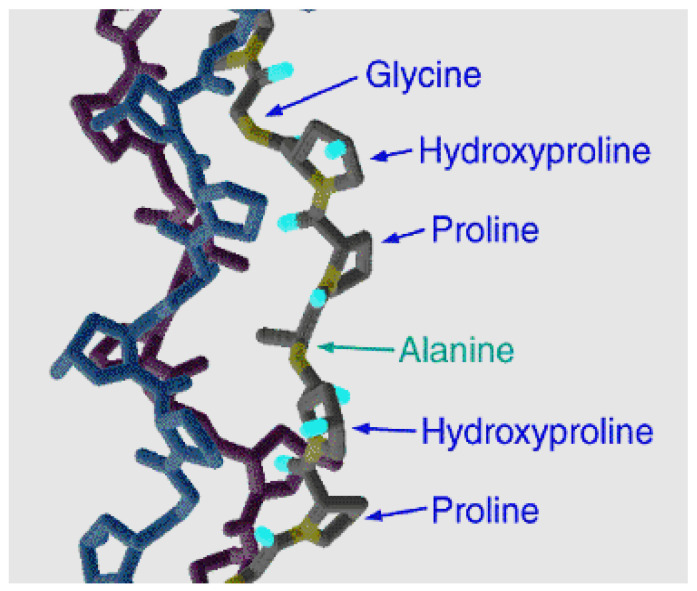
Three polypeptide α-chains form a triple-helix of gelatin. Gelatin is stabilized by the formation of covalent cross-links, both within the gelatin triple helix and between gelatin helices.

The structure and properties of collagen and gelatin have been studied repeatedly over the past few decades; (see, in particular, [20,21,32,33,34,35,36,37,38,39,40,41,42,43,44,45,46,47,48,49,50,51,52,53,54,55,56,57,58,59,60,61,62,63,64,65,66,67,68,69,70,71,72,73,74,75,76,77,78,79,80,81,82,83,84,85,86,87]); the total number of works devoted to them is at least several thousand, and, in this article, there is no possibility even to simply quote them. It has long been established by means of electron microscopy that the diameter of gelatin macromolecules is 1400 pm, while their length is 285,000 pm [39]. These values are in good agreement with similar data found in [32] on measurements of light scattering and the viscosity of gelatin solutions, as well as with later data presented in works published in the 21st century [49,50,51,52,53,54,55,56,57,58,59,60,61,62,63,64,65,66]. Summarizing numerous data in the literature concerning the molecular structure of gelatin, it can be argued that its molecules, as well as collagen molecules, are sharply asymmetric and anisometric [32,33,34,35,36,37,38,39,40,41,42,43,44,45,46,47,48,49,50,51,52,53,54,55,56,57,58,59,60,61,62,63,64,65,66]. The molecules of these macromolecular compounds consist of three polypeptide chains with almost the same molecular weight, two of which are usually almost identical to each other in the set and sequence of amino acids (the so-called α1-chains), while the third (the so-called α2-chain) differs from the other two in this respect [32,42]. The typical stoichiometric composition of collagen, expressed in the number and range of α-chains contained in its macromolecule, is (α1)_2_(α2), rare (α1)_3_ [35]. Each of these chains contains about 1020 amino acids [35,66]. These three α-chains (α1, α1, α2) wrap around each other with the so-called “single-residual” displacement, forming a right-handed triple helix [35,53,56,57,66]. A schematic representation of the polypeptide chains that make up gelatin molecules is shown in Figure 2.

A unique feature of gelatin, which distinguishes it from other natural polypeptides, is the surprisingly strict regularity of the arrangement of amino acid residues in the α1-chain: starting from the 17th amino acid residue, glycine (GLY) invariably occupies the third position so that the general formula of the polypeptide unit of the α1-chain can be written as GLY-A-B, where A and B are any amino acid residues. It is also characteristic that the second most common component in the peptide set of the gelatin molecule, proline, is almost always in position A, and its close analog hydroxyproline is almost exclusively in position B. In addition, the GLY-PRO-HYP tripeptide link in the α1-chain is the most common and occurs in more than every tenth case (39 times out of 337 possible) [37]. As for the sequence of amino acid residues in the α2-chain, as far as is known, it still cannot be considered fully established [35,53,56,57,66]; in any case, we did not find information on this subject in the known literature.

The high content of proline and hydroxyproline, as well as the curious fact that, according to the data of chemical analysis of the sequence of amino acid residues, every third residue is GLY, has long ago led the authors [20,33] to two alternative assumptions regarding the geometry of (α1)_2_(α2)- or (α1)_3_—structures of gelatin molecules, in each of which the formation of the above triple helices is postulated. Both structures proposed by them consist of three parallel polypeptide α-chains twisted into a “left” helix with proline and hydroxyproline residues in a specific *trans*-conformation. In both these structures, the helical chains are stabilized by interchain hydrogen bonds –(–)NH…O=C(–)–. However, in the structure proposed in [20], one such bond is required for every three amino acid residues, while in the structure of [33], twice as many of these bonds are needed. Despite this difference, an unambiguous choice between these two alternative structures turned out to be impossible even after X-ray diffraction analysis. Additionally, although various independent physicochemical methods of analysis have been repeatedly proposed to solve this problem, the question of the exact structure of gelatin in connection with the above theoretically permissible possibility still remains debatable. Each of the peptide fragments of the gelatin molecule is characterized by the conjugation of the π-electrons of the C, N, and O atoms, as a result of which the entire group (–C–C–NH–C(=O)–) acquires a quasi-planar structure. The carbon–nitrogen interatomic distance in this structural fragment is 132 pm, which is much shorter than the C–N single bond length (147 pm), and hence, this bond is very close to a double bond in terms of the degree of multiplicity.

**Figure 3:**
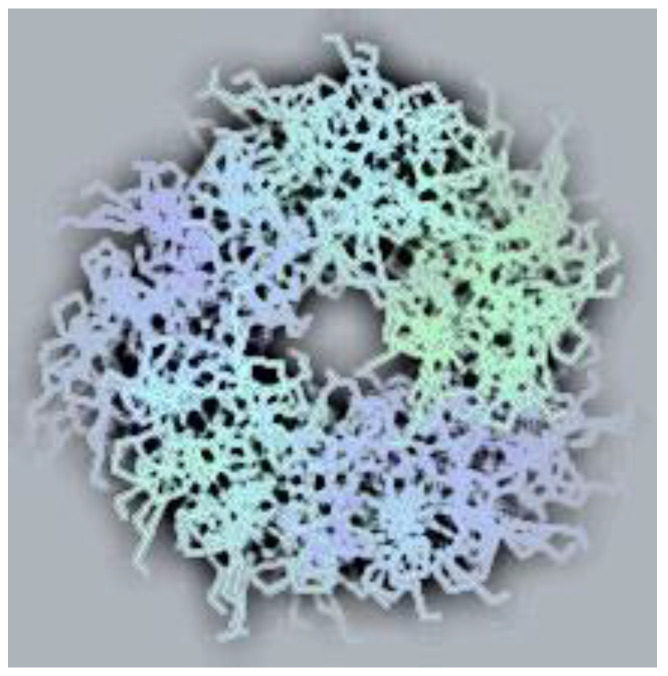
Coils and nets in the gelatin structure.

As mentioned above, during the transformation of collagen into gelatin, a polydisperse mixture of fractions with different structures and molecular masses is formed. The composition of this mixture includes single (α1 and α2), double (β11 and β12), and triple (γ) polypeptide chains, which are formed as coils or clots, as a result of which gelatin acquires a mesh structure, a stylized image of which is shown in Figure 3. In terms of their elastic properties, gelatin masses are similar to rubber; this is quite natural for infinite networks formed by long rows of molecules and interconnected by a limited number of cross-linking molecules [46,48,49]. Such a structure should have a high degree of flexibility and elasticity, and potentially it is very convenient for the immobilization of a wide variety of substances with their fixation due to intermolecular interactions; on the one hand, it does not allow any rigid crystalline blocks to be realized, on the other hand, it has a sufficiently large number of cells for the receiving and subsequent “fixing” of molecules in the immobilized substance. In addition, these cells, already filled with molecules of a chemical compound, retain a certain freedom of movement in space. One can try to estimate the size of such a cell [67]. Indeed, the volume of the polymeric gelatin layer (V_e_) with an area of 1 cm^2^ and a thickness of 20 µm is equal to (1.0 × 1.0 × 20 × 10^−4^) cm^3^ = 2.0 × 10 g/cm^3^, which is (0.5 × 2.0 × 10^−3^) g = 1.0 × 10^−3^ g. Since, as we mentioned earlier, the molecular mass of gelatin (*M*_Gel_) is ~(2.0–3.0) × 10^5^, the number of its molecules in a mass equal to 1.0 × 10^−3^ g would be (1.0 × 10^−3^/*M*_Gel_) × *N_A_*) = (1.0 × 10^−3^/*M*_Gel_) × 6.02 × 10^23^) = (2.0–3.0) × 10^15^. It has already been noted above that a gelatin molecule has an average length of 2850 nm and a width of 14 nm; assuming that it can be likened to a narrow band cylinder, its volume *V_M_* is (1/4)πD^2^h = (1/4) × 3.14 × (2850 × 10^−8^ cm) × (14 × 10^−8^ cm)^2^ = 4.38 × 10^−19^ cm^3^. With the densest packing, these molecules occupy a total volume that is equal to (4.38 × 10^−19^ × (2.0–3.0) × 10^15^) = **(8.76–13.15)** × **10^−4^** cm^3^. It can be postulated that the volume of void cells of interest to us is equal to the total volume of the polymer array minus the volume occupied by the gelatin molecules (2.0 × 10^−3^ − (8.76–13.15) × 10^−4^) cm^3^, which we have just calculated to be **(0.69–1.12)** × **10^−3^** cm^3^. Then, the average volume of the cell is found as a quotient by dividing their total volume by the number of gelatin molecules and, as you can easily see, results in **(3.7–5.6)** × **10^−19^** cm^3^. The linear size of such an “averaged” cell, assuming a spherical shape for it, would be equal to D = (6V/π)^1/3^ = (6 × (3.7–5.6) × 10^−19^ cm^3^/3.14)^1/3^ = **(8.91–10.22)** nm, assuming a cubic form. The numbers in the graph add up to more than 100, which is equal to a = V^1/3^ = ((3.7–5.6) × 10^−19^ cm^3^)^1/3^ = **(7.18–8.24)** nm. As can be seen from these values, with such cell sizes, rather large molecules of the immobilized substance can be introduced into it. The relatively large gaps between the chains of the spatial network in the molecular structure of gelatin allow molecules and ions of low molecular mass substances, unlike large colloidal particles or macromolecules, to diffuse into its intermolecular voids almost as easily as into liquid-phase solvents. At the same time, gelatin systems (both thin-layer and thick-layer ones) also have high transparency and plasticity. These properties make them very convenient for study by various spectroscopic methods (first of all, spectroscopy using the UV, visible, and near-IR spectral regions). It is also very important that gelatin arrays are quite easily destroyed under the influence of various proteolytic enzymes (trypsin, *Bacillus mesentericus*, *Bacillus subtilis*, etc.). Due to this, the chemical compounds immobilized in it can be rather easily isolated from it in the form of solid phases and analyzed by the same modern physicochemical methods as solid substances isolated from liquid-phase or gas-phase reaction systems.

**Figure 4:**
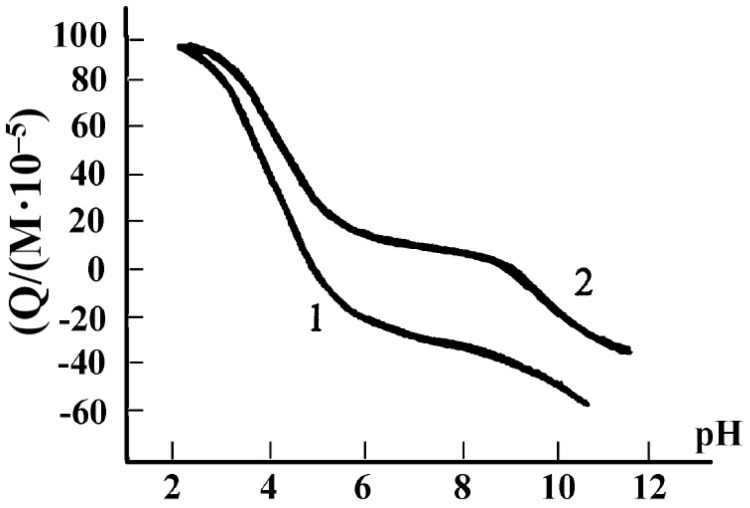
Dynamics of changes in the total electric charge of gelatin macromolecules in terms of a unit of molecular mass (in **(Q/(M·10^–5^)** values) depending on the pH of the medium: **1**—for alkaline gelatin, **2**—for acid gelatin. (This figure was made by the author of the given article according to the data presented in [78]).

Gelatin has a very significant surface area and a developed system of micropores [30,31,32,33,42,43,44,45,46,47,48,49,50,51,52,53,54,55,56,57,58,59,60,61,62,63,64,65,66,67]. It has a remarkable ability to form homogeneous systems (and, moreover, is transparent) with water at any ratio of the indicated ingredients. All such mixtures, taking into account the size of gelatin molecules, should be classified as colloidal solutions. In this connection, one more important feature of gelatin should be noted—the ability of its sufficiently concentrated aqueous solutions, when cooled to a certain threshold temperature, to form a very specific substance that is incapable of flow—the so-called jelly. During jellying, the viscosity of the solution gradually increases, and upon reaching the above threshold temperature (the so-called jelly formation temperature *T*_jel_), it abruptly increases to an infinitely large value. This state of gelatin has a continuous three-dimensional network structure; the statistical condition for its formation is the presence of at least one cross-link per mass-averaged gelatin molecule. It should be emphasized that the chilliness temperature *T*_chil_ and the jelly formation temperature *T*_jel_ are different parameters since the first is determined only by the nature of gelatin and have a fixed value for each of its varieties, the second also depends on the mass ratio of gelatin: water in the water-gelatin colloidal solution increases with the growth of the latter, approaching the limit of the chilliness temperature. It should be noted in this connection that the constituent parts of gelatin molecules—α-chains can be isolated by the thermal denaturation of collagen at a temperature above the chilliness temperature, upon reaching which there is a rather sharp transition from (α1)_2_α2- or (α1)_3_-structures in α-, β- and γ-chain, which is visually perceived as “melting”. However, there is no melting in the classical sense of this term since both gelatin and collagen are amorphous substances. It is characteristic that such properties as viscosity, light scattering, and optical activity when collagen is heated to *T*_chil_ change abruptly. The heat-denatured macromolecule of soluble collagen returns to the “solid” state upon cooling; however, its original structure is only partially restored [20,33,37]. The difficulty of the complete restoration of the original structure is associated with steric difficulties of the “alignment” of α-chains that are necessary for this and their subsequent ordering. Additionally, although the original gelatin consists entirely of three completely ordered polypeptide α-chains, their dissociation can and actually does lead to the appearance and preservation of a large number of “imperfect” structures upon cooling below *T*_chil_. Only γ-gelatins with natural [34,68] or artificial [69] cross-links are capable of the complete restoration of the original structure, and the rate of such a process is usually much higher than the same parameter for single (α) or double (β) gelatin chains [70]. It is noteworthy that the *T*_chil_ value of gelatin may be changed both upwards and downwards by adding small amounts of a number of substances to it; thus, sodium salts with inorganic anions shift it from +4.5 °C (in the case of NaF) to −14.0 °C (in the case of NaSCN or NaI) [37]. Organic compounds, in particular, carbamide, carboxylic acids, and guanidinium salts can also have a rather strong influence on this parameter. At the same time, changes in the *T*_chil_ values under the action of salts of various metal ions are largely determined by the ability of gelatin to restore its original structure [71]. It should be expected theoretically that such parameters which associated with the process of gelatin gelatinization as *T*_chil_. and the time of chilliness *t*_chil_., should depend on the size of its macromolecules, the nature of the branching of α-chains, and the chemical nature of their constituent polypeptide units. However, no reliable and serious correlations in this regard have been found so far. Only the fact of the existence of a certain critical molecular mass (*M* ~ 100.000) was established, below which the strength of the jelly (G) depends quite strongly on *M* [72]. The pH value, as well as the ionic strength of the water-gelatin solution, has a significant effect on the strength of the jelly [73]. In particular, it has been observed that the *G* values of the gelatin jelly undergo a sharp decrease upon reaching pH < 5 and pH > 10. The authors of [74] associated the adverse effect of both acidic and alkaline solutions on the formation of gelatin jelly with the high total charge acquired by gelatin macromolecules in such solutions, which contributes to their repulsion from each other and thereby contributes to the “unfolding” of the collagen structure and the formation of “random” coils from these macromolecules. The appearance of such a charge on gelatin macromolecules is not surprising—gelatin, in accordance with the Brønsted-Lowry protolytic theory [75,76,77], is a typical ampholyte because it contains acidic and basic groups that are capable of quantitative titration in aqueous solution, such as β-carboxylic aspartic acid, β-carboxylic glutamic acid, β-imidazole histidine, ε-amine lysine, δ-guanidine arginine, and phenol-hydroxyl tyrosine. Additionally, although gelatin is an electrically neutral compound, it actually exists in aqueous solutions in the zwitterionic form, so it is quite natural that it has a so-called isoelectric (isoionic) point (p*I*), in which the positive charges of the terminal NH_3_^+^ groups are neutralized by the negative charges of the terminal COO^–^ groups. The alkaline gelatin’s p*I* is in the range of pH = 4.8–5.1; it is characteristic that at the pH value corresponding to it, all the main groups carry a positive charge, and gelatin molecules contain the same number of negative charges due to the deprotonation of most (though not all) carboxyl groups. For acid gelatin, the range of possible p*I* values is much larger (7.0–9.5); in this case, all carboxyl groups are deprotonated, and their charge is balanced by the positive charge of guanidine and most ε-amino groups. It is noteworthy that the p*I* value for the “progenitor” of gelatin, collagen (9.0–9.5), also falls into the same range. A typical example illustrating the nature of the change in the value of the total charge of alkaline and acidic gelatin depending on the pH is shown in Figure 4. As can be seen from it, both of these gelatin types have a significant positive charge at pH < 4.0 and noticeably smaller negative charge at pH > 10.0, while in the range pH = 5.0–9.0, their charges are opposite [78]. As already mentioned above, gelatin is a mixture of substances (moreover, differing in their electrical charge), and therefore, the measured pH value of the isoelectric point is an average of values that differ from each other up to several pH units.

**Figure 5:**
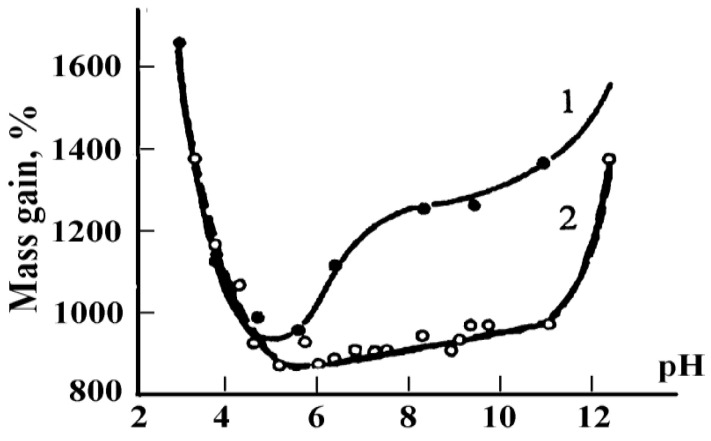
Gelatin swelling curves depending on pH: 1—for alkaline gelatin, 2—for acid gelatin. (This figure was made by the author of the given article according to the data presented in [79]).

Owing to the developed surface with a system of micropores, when gelatin came into contact with any aqueous solution, both the solvent molecules and molecules of substances dissolved in it penetrated into its array. The introduction of water molecules into the voids between the α-chains of gelatin macromolecules led to such an important phenomenon from a practical point of view regarding its swelling that its absence would mean that the diffusion of dissolved substances from any solution in contact with this polymer mass would be very difficult. As far as gelatin “imbibes” more and more portions of the solvent, the polypeptide links between the cross-links become more and more elongated; this process stops when these links lose their flexibility. Quantitatively, the degree of swelling can be defined as the ratio of the mass of the polymer array resulting from the absorption of the solvent into the initial mass of this array. The degree of swelling for gelatin depends on the pH. As the experimental data show, the nature of such dependence for acid and alkaline gelatin is not the same: for alkaline gelatin, a fairly well-defined minimum is observed, located near the isoelectric point, while for acid, it is strongly “smoothed” (Figure 5). Comparing the data of this dependence with the data of Figure 4, it is easy to note a clear correlation between the degree of swelling and the total charge in the gelatin macromolecule at the corresponding level of acidity. The presence of various ions, both cations and anions, in the aqueous solution that are in contact with the gelatin mass greatly contribute to swelling; according to the degree of such, they are located in the so-called Hofmeister’s lyotropic series [80,81], whose fragments for cations and anions, in particular, are the series Ca^2+^ > Sr^2+^ > Ba^2+^ > Mg^2+^ > Na^+^ ~ K^+^ and SCN^–^ > I^–^ > Br^–^ > Cl^–^ > SO_4_^2–^. The influence of dissolved substances on the swelling of gelatin is associated primarily with the destabilization of individual sections of the collagen structure and their aggregates [82]. It should be noted in this connection that the position of solutes in the lyotropic series can change depending on their concentration in a solution [82,83,84,85,86]; in particular, some salts at some concentrations, to some extent, prevented swelling compared to that for a pure solvent, while others, on the contrary, enhance it [84,85,86]. This circumstance, in principle, may well affect the kinetics of a number of processes involving gelatin arrays.

In addition to acid-base properties, gelatin also has the ability to act as a polydentate chelating ligand in complexing processes and as a reducing agent in redox processes. Both of these functions of gelatin are associated with the presence of peptide groups (–C–NH–C(=O)–C–) in its molecular structure. The first of these processes, in this case, is very complex and ambiguous if only because, in this case the formation of both the homolig and metal is observed.

Complexes (containing only one protein molecule as a ligand in the inner coordination sphere) and heteroligand ones (containing either two different proteins or a protein and any of those chemical compounds that are contained in gelatin as impurities in the inner coordination sphere; for example, sodium thiosulfate appears in the process of the industrial production of gelatin). In this regard, it should be noted that the complexing of gelatin with Cr(III) cations, as well as Ti(IV) and Zr(IV) cations, underlies a practically important process called “mineral tanning”. The second of the above processes usually occurs when gelatin is exposed to sufficiently strong oxidizing agents; these include, in particular, potassium permanganate, potassium dichromate, and elemental iodine. On the other hand, the reducing properties of gelatin are also associated with its presence in various impurities of both a natural and anthropogenic origin. More detailed information on the chemical properties of gelatin can be found in the monograph [87].

Among the applications of gelatin and its modifications, which are associated specifically with its micro- or nanostructural organization, the following are included:

- The production of silver halide photographic materials for recording information (using so-called photographic gelatin);
- The creation and immobilization of nanoparticles of various chemical compounds;
- The creation of pharmaceutical dosage forms—as a material for the manufacture of capsules, components of nutrient mixtures and media, as well as a component of plasma-substituting and diagnostic tools;
- The creation of protein-based nanomaterials.

The choice of the first of the above areas for consideration in the given review article is due to the fact that the use of gelatin in this capacity is the historically earliest use of it, which was based precisely on the chemical properties of this biopolymer. The second field of its application has actually become a logical continuation of the first one since, firstly, when creating many gelatin-immobilized systems, it was silver halide photographic materials that were used as the “raw material”, and secondly, it turned out that chemicals contained in such immobilized systems had a nano-structural level of organization. The third field of application of this protein is currently the most in-demand in practice, primarily in medicine, and therefore deserves the closest attention since we are talking about the treatment of a wide variety of human diseases. Finally, the last of the above options for using gelatin is associated with obtaining it in the form of nanoparticles, which, in turn, can be involved in various nanotechnologies. It is these four areas that make up the entire array of possible applications of this biopolymer, associated precisely with its specific physicochemical and structural characteristics; each of these areas will be further developed in its own special section. Let us now consider these applications in the order listed above.

## 4. Gelatin as a Binding Agent for Recording Systems in the Silver Halide Photographic Process

The use of gelatin in this capacity is historically the earliest of its uses, which is based on the chemical properties of this biopolymer. The silver halide photographic process, as well as the use of gelatin in it, dates back to 1871; it was then the English medical advisor R.L. Maddox unexpectedly discovered that if firstly silver nitrate and then—potassium bromide or potassium iodide are added into a heated gelatin solution, then the “photosensitive liquid” prepared in this way (which subsequently received the not quite correct name “photographic emulsion”), has a sensitivity visible light many times higher than any of the other photosensitive compositions known at that time [88]. Namely, he first proposed the use of the preparation of such a “photosensitive liquid” gelatin obtained from the bones and skin of cattle, which swelled well in cold water and became permeable to aqueous solutions. The materials prepared as a result of such a process have been the dominant materials of information registration for more than a hundred years, which was facilitated by an exceptionally successful combination of its main “actors”—silver halides AgHal (where Hal is Cl, Br, J) in certain quantitative combinations as a component sensitive to visible light quanta (as well as some other types of electromagnetic radiation) and gelatin as a binder, which plays a very important role in the specific chemistry of this process. Without going into details here, we would like to note that the key product of this information recording process was a photographic image obtained during the catalytic reduction of AgHal to elemental silver when treated with special solutions, collectively called “developers”. This catalytic process occurred on elemental silver nanoparticles when formed during the photolysis of AgHal—the so-called development centers; the rate of the AgHal→Ag reduction process on such particles is several orders of magnitude higher than the rate of reduction of silver halides in those places where these particles are absent. This process is described in detail in a number of books and monographs and is worth highlighting [89,90,91,92,93]. The components of this image were elemental silver (the so-called “building material” or “carrier”) and gelatin as a binder substance (the so-called “binder”). In many cases, namely this image (called “silver” by the epithet) was the end product of the silver halide photographic process; one variety of this process, accompanied by its formation, is called “black-white photography”. In this connection, it should be especially noted that numerous attempts to replace gelatin with some other natural or synthetic polymer have failed for at least two reasons. Firstly, it was precisely when using this binder that a sufficiently good adhesion of the photosensitive layer of the silver halide photographic material to a wide variety of surfaces (substrates)—glass, metal, cellulose, etc. was ensured. Additionally, and this is the main thing, gelatin contains a number of catalytically active impurities, including sulfur-containing compounds with the so-called labile sulfur, one part of which is “inherited” from its “progenitor”—collagen (in particular, cystine and cysteine, albeit in small quantities)—the other appears as a result of processes of transformation from the collagen into gelatin [89,90]. Additionally, the high sensitivity of the silver halide photosensitive material with a gelatin binder to electromagnetic radiation quanta is determined not so much by the amino acid set of gelatin molecules as by these same impurities. For example, the presence of impurity sulfur-containing compounds in gelatin leads to the fact that, along with elemental silver, small amounts of silver sulfide (Ag_2_S) nanoparticles are also included in the development centers, due to which the catalytic activity of these centers in the development processes further increases compared to that for the centers manifestations that do not contain Ag_2_S. The specifics of gelatin as a photosensitive material in various versions of the silver halide photographic process are also considered in detail in a recently published review article [91].

Black-white photography, however, was the earliest variant of the silver halide photo process. Later, other varieties of it appeared, as a result of which monochrome and polychrome photographic images, the “building material” in which were various inorganic and organic substances with fairly intense absorption in the visible region of the spectrum, were obtained. The earliest among these varieties were the processes of toning, during which images were obtained, which, along with elemental silver, usually included colored inorganic compounds. Within the framework of such processes, either the partial or complete transformation of elemental silver into insoluble colored and hardly soluble in water silver compounds or the partial replacement of elemental silver by a colored compound of another metal element (for example, cobalt, nickel, copper), or simply the adsorption of organic dyes on the surface of elemental silver microcrystals took place. A number of such processes are described, in particular, in monographs [92,93,94]. However, the most widespread among them was “color photography”, in which various organic dyes were the carrier of the photographic image; this process is described in detail in the monographs [89] (Chapter 17) and [90] (Chapter 12) cited above, as well as in [92,93,94,95,96,97,98,99,100]. This process is interesting because the end product was a non-silver photographic image, i.e., the elemental silver contained in the initial photographic material was completely removed from it during its processing and accumulated in one of the processing solutions. Despite a number of difficulties associated both with the manufacture of silver halide materials for color photography and with their processing (which, as a rule, had to be carried out in complete darkness), this type of silver halide photographic processing has gained quite considerable popularity.

At the end of the 70s of the 20th century, in the industry that was associated with the production of silver halide photographic materials, due to the shortage of silver that had already begun at that time, the problem of all-around saving this precious metal arose with all acuteness. This problem was exacerbated by the circumstance that the photographic industry at that time consumed almost 30% of all silver mined in the world. The circumstance noted above has made popular such photo processes on silver halide photographic materials, which were aimed at decreasing the content or even completely replacing this precious raw material in photographic images obtained as part of a black-white photographic process. This role could, in principle, be claimed by the color silver halide photo process; however, an unpleasant circumstance was revealed: the preservation of the color images obtained with its participation was poor due to the organic dyes that form then relatively quickly “fade” under the action of both light and aggressive environmental agents. In this regard, processes with so-called physical development were based on the idea that the three functions that AgHal performs in black-white photography, namely (*a*) a photosensitive compound, (*b*) a compound from which centers of development are formed, (*c*) “building material” for forming a photographic image, would be distributed between two or even three different chemical compounds. In general, functions (*a*–*c*) can be displayed with stylized schemes (1)–(3), respectively

A + hν→B(1)

B + C→D(2)

M^z+^ + Red→M + Ox (3)

where A is a light-sensitive compound, B is a product formed by the action of radiation on A, C is a substance that forms catalyst D upon reaction with substance B, M^z+^ and M are reducible metal ions and their reduction product, and Red and Ox are a reducing agent and its product oxidation, respectively. In this case, reaction (3) must be catalyzed by both substance D and elemental metal M, i.e., be autocatalytic. In such a variant, the so-called low-silver photographic materials, the content of AgHal (and, accordingly, silver), which is much less than in traditional silver halide photographic materials, are present since here AgHal is needed only to form development centers, and nothing more. To implement this process, various reactions of chemical precipitation of metals from aqueous solutions can be used, which, however, must be kinetically retarded and proceed at a low rate in the absence of a catalyst; be catalyzed by metal particles formed during a photochemical reaction in the photographic layer (or upon contact of the exposed layer with a solution containing reducible metal ions); be autocatalytic in nature, i.e., be catalyzed by the product formed at the development centers. As a result, photographic non-silver images consisting of various elemental metals that were cheaper than silver—cobalt, nickel, copper, etc.—with a high degree of dispersion and very high optical densities, even with their low content in the resulting images, were obtained. At one time, a number of so-called physical developers existed in which such a process was realized; these processes, however, were mainly described either in the patent literature or in journals that are not very accessible to modern researchers. A list of some publications devoted to these specific systems is presented in the review article [101]. Although at different times processing solutions were also proposed to the realization of the above idea, which made it possible to obtain photographic images from other elemental metals on low-silver photographic materials, they did not receive significant practical application due to the fact that after even a single use and further storage, they very quickly lost their efficiency. That much is known to the author of this article, but no one of the researchers was able to solve this problem. On the other hand, it turned out that photographic images formed by elemental metals, which are more active than silver, also did not show good retention over time. Somewhat more promising was the process of enhancing black and white silver images by the so-called “re-precipitation” of elemental silver [102,103,104,105,106,107,108,109], in which a silver image was again obtained on low-silver photographic materials, but it had a red-brown or black-brown color and much higher optical densities compared to those for the original image. Additionally, although the processing solutions used in this process were distinguished by good storability, they remained unclaimed by consumers of silver halide materials.

On this background, a different approach to obtaining non-silver photographic images could have been developed, namely, those whose carriers are intensely colored chelate complexes of *d*-elements. In this version, a silver image obtained on commercially available photographic materials was first exposed to a special solution containing potassium hexacyanoferrate(III) K_3_[Fe(CN)_6_] and a complex of any of the 3*d*-element ions with oxalate, citrate, or tartrate anions, as a result of which elemental silver was transformed into a mixture of silver hexacyanoferrates(II) and the corresponding 3*d* element. Then, it was treated with sodium thiosulfate solution; in this case, the transition of silver hexacyanoferrate(II) into its soluble complex with the thiosulfate anion, and the complete removal of silver from the processed photographic material took place. The final stage of this process was the treatment of the photographic material with an alkaline solution containing an organic compound capable of forming a chelate complex with the ion of a given 3*d* element. This process was also quite laborious; however, the stability of both the resulting non-silver image and the processing solutions within its framework was significantly higher than in those within the framework of the process with physical development. Attempts to obtain such images were made as early as the early 1980s, but the first significant publication on this subject (in the form of an article in the journal [110]) appeared only at the end of 1989. There is mainly literature that is patent on this issue; among the few publications in the form of articles in journals, one can mention [110,111,112,113,114] and reviews [115,116]. This process, however, also did not find wide practical application; practicing photographers were confused by its duration (which took at least 1 h in total) and even by the unusual nature of the substances consisting of non-silver images. At present, due to the rapid development of digital technologies, the silver halide photographic process has receded into secondary roles, and although, to a certain extent, it still retains its importance in such areas as industrial and medical X-ray diagnostics, there is no doubt that in the future it will be of only historical interest.

## 5. Gelatin as a Matrix for the Creation and Immobilization of Nanoparticles of Various Chemical Compounds

To some extent, this paragraph can be considered as a continuation of the previous one because, strictly speaking, the chemical reactions that occur within the framework of the silver halide photographic process are nothing more than reactions in silver halide gelatin-immobilized matrices or in gelatin-immobilized matrices containing elemental silver.

In the general case, the concept of “immobilization” (lat. *immobilise*—slow, fixed, motionless) in relation to a chemical compound in the broad sense of the word means any restriction of the mobility of its molecules or their associates in the physicochemical process in which this compound participates. Quantitatively, this same mobility, both at the macro- and micromolecular levels, can be characterized by a parameter similar in its meaning to the well-known number of degrees of freedom from thermodynamics [117,118]. In a narrow sense, immobilization means the fixation of a particular chemical substance with a certain degree of rigidity on the surface or in the volume of another substance in a solid state of aggregation (the so-called carrier); the role of the latter, as a rule, is some high-molecular compound (in particular, a polymer)—inorganic or organic, natural, or synthetic. The objects formed as a result of such a fixation, and also, as a result of the participation of immobilized substances in any chemical transformations or under the physical influence on them, received the collective name of polymer-immobilized systems (although, as already mentioned above, not every high-molecular compound is a polymer). In the process of immobilization, in particular, a change in the degree of steric “availability” of molecules or structural fragments of the immobilized chemical compound for contact with reactive chemical compounds occurs; as a result of this, the reactivity of such a compound in various chemical processes changes and it becomes possible to purposefully regulate it. By varying the nature of the polymer carrier, one can change not only the reactivity of a particular chemical compound but sometimes even the nature and direction of the chemical reaction in which this compound participates. In many cases, polymer arrays with substances immobilized in them are applied in the form of thin layers on any substrates, which can be formed both by inorganic (glass, ceramics, zeolites, etc.) and organic (cellulose, organic glass, polyethene terephthalate, etc.) materials. Further, we name such physicochemical objects as polymer-immobilized matrix systems, polymer-immobilized matrices, and polymer-immobilized matrix implants. General issues related to immobilization in polymer arrays and reactions involving immobilized chemical compounds are considered in monographs and review articles [117,118,119,120,121,122,123,124].

In principle, there are two variants for the immobilization of a substance—either with the use of functional groups of a molecule of a high-molecular compound (polymer) and the formation of metal-polymer bonds (which can be conditionally called “chemical immobilization”) or due to dispersion, orientation, induction and other similar types of interaction—for which only the presence of a well-developed polymer surface is sufficient (and which can conventionally be called “physical immobilization”) [117,118]. This second variant of immobilization can be carried out in a variety of ways—by dispersion, adsorption, or precipitation of the target substance in the polymer layer, sputtering, impregnation, etc. In this case, the most preferable is the deposition of the target chemical compound directly in the polymer array, which is carried out as a result of appropriate chemical transformations since it is in this case that the nano-structural level of organization of the immobilized substance can be achieved. Gelatin belongs precisely to those high-molecular compounds, the molecules of which are associated with the immobilized substance almost always only due to the physical immobilization [124] (although, of course, in principle, chemical immobilization can also take place in it, albeit to an insignificant degree). Since, as already mentioned above, gelatin molecules, depending on the pH of the solution, have either a positive or negative charge, one very important consequence follows from this: all chemical processes leading to the immobilization of metal complexes in a gelatin mass proceed when polymer macromolecules have an electric charge. Additionally, this is namely: “+” if the pH value of the reaction medium (actually determined by the pH value of the solution in contact with the polymer matrix of the solution) is below the p*I* value, and “–” if the ratio of these parameters is inverse.

The theoretical foundations of the complexing process in gelatin-immobilized matrix systems are described in the work [123]. The greatest number of publications in this direction are connected with the immobilization of metal complexes of various 3*d*-elements. In all these works, gelatin-immobilized matrices containing elemental silver were used as the initial “raw materials”, which, as part of the above process, was subjected to specific chemical processing proceeding in three stages, namely:

(1) The treatment of a silver-containing gelatin-immobilized matrix with an aqueous solution containing a complex of the corresponding 3*d*-metal ion with any organic acid (usually oxalic, citric, or tartaric), potassium hexacyanoferrate(III) (ferricyanide) K_3_[Fe(CN)_6_], and agent to create the acidity of the medium necessary for proceeding the reaction (usually slightly alkaline). As a result, elemental silver contained in the initial gelatin matrix was transformed into silver hexacyanoferrate(II) (ferrocyanide) Ag_4_[Fe(CN)_6_] and, simultaneously, the formation of hexacyanoferrate(II) of the corresponding 3d-metal ion took place in the gelatin layer of the immobilized matrix.

(2) The treatment of the matrix obtained at stage 1 with an aqueous solution containing trioxosulfidosulfate(VI) (thiosulfate) of sodium Na_2_S_2_O_3_. The silver hexacyanoferrate(II) contained in the matrix was transformed into a complex of silver with a thiosulfate anion, which is highly soluble in water and, due to this, passed from the gelatin matrix into the solution in contact with it; the 3*d*-metal hexacyanoferrate(II) deposited together with it at stage 1 did not interact with Na_2_S_2_O_3_ and remained unchanged in the immobilized matrix. As a result of this process, a 3*d*-metal hexacyanoferrate(II) gelatin-immobilized matrix was obtained.

(3) The treatment of the gelatin matrix obtained at the end of stages (1) and (2) is performed with an aqueous alkaline solution containing an organic substance that is capable of acting as a chelate ligand and forming a corresponding poorly soluble complex in water with a given 3*d*-metal ion (as a rule, a metal chelate). This gelatin-immobilized metal chelate was the final product of the synthesis.

The details of such a process were described in [110,111]. Such a scheme, however, was implemented only for a small number of metal ions, namely, Ni(II), Cu(II), Fe(III), and Co(III); for most other *d*-metal ions, two substages had to be used within step (1), namely:

(1a) The treatment of the silver-containing gelatin-immobilized matrix with an aqueous solution of potassium hexacyanoferrate(III) to convert elemental silver to silver hexacyanoferrate(II);

(1b) The treatment of the matrix obtained in substage (1a) with an aqueous solution of chloride or bromide of the corresponding *d*-metal ion. As a result of the contact of the matrix with such a solution, silver hexacyanoferrate(II) was converted into hardly soluble silver chloride AgCl or silver bromide AgBr, whereas the metal ion was bound to the hexacyanoferrate(II) anion [Fe(CN)_6_]^4–^, turning into the corresponding hexacyanoferrate(II).

Such a method of obtaining gelatin-immobilized hexacyanoferrates(II) (which, as is easy to see, were the initial metal-containing precursors for various complexation processes) is universal and, in principle, is suitable for any metal ions.

The processes associated with the immobilization of chemical compounds in a gelatin matrix have been considered in a very significant number of original articles, among which we should first of all mention [125,126,127,128,129,130,131,132,133,134,135,136,137,138,139,140,141,142,143,144,145,146,147,148,149,150,151,152,153,154,155,156,157,158,159,160,161,162,163,164,165,166,167,168,169,170,171,172,173,174,175,176,177,178,179]. Very likely, the largest number of works in this direction was associated with the immobilization of metal complexes; the earliest of them should be considered [125], in which the immobilization of the Ni(II) chelate complex with the deprotonated form of dithiooxamide (ethandithioamide) H_2_N–C(=S)–C(=S)–NH_2_ was carried out. This article gave impetus to further research in this direction, as a result of which the list of gelatin-immobilized metal complexes began to grow rapidly. The given objects now make up the majority of gelatin-immobilized chemical compounds; as a rule, these are compounds formed by such 3*d* elements as Co, Ni, and Cu. At first, these were mainly metal chelates obtained under the conditions of classical complex formation (nucleophilic substitution), i.e., as a result of interaction between a metal ion and some organic compound acting as a ligand [125,126,127,128,129,130,131,132,133,134]. In particular, there was carried out an immobilization in a gelatin matrix of such known metal chelates as Ni(II) with dimethylglyoxime [132], Cu(II) with dithiooxamide and its substituted ones [126,128,131], and Co(III) with 8-mercaptoquinoline [130]. At the turn of the 20th–21st centuries, template synthesis reactions were also added to the list of processes used for immobilization in the given biopolymer [135,136,137,138,139,140,141,142,143,144,145,146]. In this regard, it should be specially noted that in a number of cases, these reactions produce specific chemical compounds, the formation of which does not take place under the traditional conditions for the implementation of the same reactions (i.e., in solution or solid phase) (see, for example, [125,138,140,142]). A detailed consideration of these specific processes, however, is already beyond the scope of our narrative; review articles are devoted to them [123,147,148,149,150,151,152,153,154,155]. The theoretical foundations of the complexation process in gelatin-immobilized matrix systems are described in [156]. In addition to obtaining gelatin-immobilized metal chelates, the literature at different times considered the possibility of immobilization in this biopolymer of other chemical compounds, both inorganic, namely hexacyanoferrates(II) [157,158], metal sulfides [159,160,161], and organic, acid–base indicators [162,163,164,165,166]. A fairly significant number of works are devoted to gelatin-immobilized proteases (see, in particular, [167,168,169,170,171,172,173,174]); there is a review article on this subject [175]. The process of obtaining gelatin-immobilized elemental silver was previously described by our group [176,177,178].

The question of what level of organization of gelatin-immobilized compounds remained open until the middle of the second decade of the 21st century, although, taking into account the data concerning the internal structure of gelatin arrays, namely the size of voids in its molecular structure, one could a priori expect that as a result of this very immobilization, nanoparticles would have been mainly formed. Experimental evidence of this fact, concerning gelatin-immobilized metal sulfides, elemental metals, and metal complexes, appeared only in the mid-10s of the 21st century; the corresponding data, namely those obtained by scanning electron microscopy (SEM), are given in the publications [67,161,178] cited above, as well as in the recently published article [179]. In this connection, there is every reason to believe that gelatin-immobilized matrix systems formed as a result of various physicochemical processes, at least in most cases, with a nano-structural level of organization of chemical compounds immobilized in them.

## 6. Gelatin as a Matrix for the Creation and Delivery of Pharmaceutical Drug Forms

It is well known that the determining role in the therapeutic system and in the traditional dosage form belongs to the carrier in which the drug is enclosed. It is on this carrier that the speed and completeness of the release of the active substance of the drug in the body depends, and, consequently, the degree of its therapeutic effectiveness. An ideal carrier must meet a number of requirements: the absence of toxicity and allergenicity, its biodegradability in the body or excretion from the body unchanged, a high capacity with respect to most drugs, the accumulation of the drug at the site of action and its release at the site of action in a therapeutic dose, ensuring the protection of the medicinal substance from destruction during transport to the site of action, the possibility of long-term storage, the method of introduction into the body that is not traumatic and, if possible, the ease of manufacture, economic availability. Naturally, it is difficult to expect that real carriers will be able to meet all the requirements (if only because the range and properties of medicinal substances are very diverse). Howbeit, gelatin is a very convenient medium for creating and transporting various drugs to the human body since, on the one hand, it is a hydrophilic high-molecular compound and one of those protein products that are produced during human life; on the other hand, it easily forms very strong jellies into which both macro-, micro-, and nanoparticles of various substances can be implanted.

A very large proportion of the gelatin used in the pharmaceutical industry is realized for the manufacture of hard and soft gelatin capsules (soft gels), as well as for tableting, tablet coating, granulation, encapsulation, and microencapsulation. The encapsulation of drugs seems to be very appropriate in cases where it is required to eliminate or minimize the bad (bitter) taste and/or smell of drugs and protect them from exposure to light or aggressive environmental agents (in particular, atmospheric oxygen) [180]. Owing to it also makes possible the controlled and/or directed release of bioactive molecules contained in gelatin capsules [181,182]. For the encapsulation of drugs in such capsules, it may be useful to cross-link gelatin polymer chains in order, on the one hand, to reduce its solubility in body fluids and on the other hand, to provide a prolonged release of the encapsulated drug [183,184].

As is known, one of the most important problems of modern pharmacology and medicine is the targeted delivery of drugs to tissues and internal organs; this problem is especially important in the field of diagnostics and the treatment of oncological diseases [185,186]. Such a targeted delivery to a specific tissue/organ, on the one hand, provides a higher therapeutic efficacy, on the other hand, significantly reduces side effects and overall toxicity. A number of works were devoted to research in this direction, among which it should be noted [187,188,189,190,191,192,193] related to the diagnosis and treatment of cancerous tumors; there are also review articles on this subject [194,195]. Thus, Magadala and Amiji [187] made the first attempt to introduce the epidermal growth factor receptor (EGFR) recognition sequence into a gelatin base to study gene delivery to pancreatic cancer cells. In this work, it was shown that EGFR-modified gelatin exhibited minimal cellular cytotoxicity with significantly increased transgene expression efficiency compared to the control gelatin or gelatin nanoparticles modified with polyethylene glycol, which is traditionally used to modify this biopolymer. EGFR is known to be highly expressed on various cancer cells, and the level of expression can directly correlate with the stage of a cancerous tumor [196]; that is why it is a key target in the delivery of drugs to organs affected by a cancerous tumor [187,188,190]. In light of these promising results, biotinylated EGF-gelatin (b-EGF-gelatin) nanoparticles have been used as carriers for the targeted delivery of such a well-known chemotherapy drug as cisplatin [190]. In particular, it has been shown that cisplatin implanted in b-EGF-gelatin has a significantly higher antitumor activity compared to both individual cisplatin and unmodified gelatin nanoparticles “loaded” with this therapeutic agent [190]. Additionally, it should be noted the work [191] shows the possibility of targeted drug delivery based on gelatin across the blood–brain barrier. The specific properties of gelatin, in particular the flexibility of its molecules, were used in [192] for the highly selective targeting of cells in several organs.

In recent years, there has been a clear trend in medical practice towards the use of targeted drug delivery based on gelatin modified with various functional groups, as well as composite materials with embedded gelatin carriers. The chemical modification of the gelatin structure allows for improved drug stabilization and the increased efficiency of drug uptake by the carrier. A number of publications [197,198,199,200,201,202,203,204,205,206,207,208,209,210,211,212,213,214,215,216,217,218,219,220,221,222,223,224,225,226,227,228,229,230,231,232,233,234,235,236,237,238,239,240,241,242,243,244,245,246,247,248,249,250,251] are devoted to research in this direction, where gelatin composites are proposed for transporting drugs, containing, alongside gelatin, some other substances—including natural and synthetic, inorganic and organic. The earliest examples in this regard, apparently, are composites containing, along with gelatin, inorganic compounds, the natural mineral hydroxyapatite Ca_5_(PO_4_)_3_(OH) [197,198,199,200,201,202,203], which was first described as such 20 years ago [197], and similar compositions, such as a synthetic trisubstituted calcium orthophosphate Ca_3_(PO_4_)_2_ [204,205,206,207,208]. However, high-molecular compounds (in particular, polymers) of natural and synthetic origin have become more widely used as components for combination with gelatin [209,210,211,212,213,214,215,216,217,218,219,220,221,222,223,224,225,226,227,228,229,230,231,232,233,234,235,236,237,238,239,240,241,242]. Of natural compounds, chitin and chitosan [209,210,211,212,213], silk [214,215,216], and hyaluronan [217,218,219,220,221,222,223] are mentioned in the literature; among synthetic compounds, this included poly(lactic-co-glycolic) acid [224,225,226,227,228,229], oligo(poly(ethylene glycol) fumarate [230,231,232,233,234,235,236] and poly(propylene) fumarate [237,238,239,240,241,242]. Of the recent works, it should be noted that publications [243,244,245,246,247,248,249,250,251,252,253], along with the named components of gelatin systems, also used some other polymers, for example, methacrylate [244], polycaprolactone [246], and polypyrrole [248]. To the use of these polymers in medical practice, a review [253] was devoted. Taking into account the recent advances in targeted delivery with gelatin-based systems, it is safe to say that gelatin is expanding as a delivery vehicle, especially in the field of cancer treatment. In general, it can be stated that the modification of gelatin and its combination with other biomaterials demonstrates the flexibility of this biomaterial and ensures its important role as a carrier in the field of drug delivery and tissue engineering [194].

## 7. Gelatin as an Object for Creating Protein Nanomaterials

Protein molecules, in general, and gelatin, in particular, are actually nothing more than natural nanoparticles that have all the properties inherent in nanosystems and, in addition, specific biological functions that make them very attractive for the implementation of nanotechnologies. Obtaining nanomaterials, in this case, is based on the principles of self-organization of biostructures. The key point here is the so-called “thermodynamic hypothesis” of the folding of protein molecules, which was expressed at the time by an outstanding American biochemist and Nobel Prize winner in chemistry in 1972, C.B. Anfinsen [254]. According to his hypothesis, the original or natural conformations of proteins are obtained because this form is thermodynamically the most stable in the intracellular environment; that is, the protein molecule takes this form as a result of the restriction of peptide bonds and changes in the form by other chemical and physical properties of amino acids. The point is that proteins with a non-repeating sequence of amino acid residues in the polypeptide chain have a clearly defined (and, moreover, compact) globular conformation, which is necessary for the implementation of their biological functions. Additionally, although in gelatin molecules, as well as in other natural polypeptides, in general, there is also a non-repeating sequence of amino acid residues in the polypeptide chain, nevertheless, some regularity is still observed since, as already mentioned above, its stoichiometric composition can be displayed in a stylized formula (GLY-A-B)_n_. In this regard, it should be noted that gelatin, although it does not belong to globular to fibrillar proteins, has a structure that combines the motifs of both globular and fibrillar structures (with the dominance, however, of fibrillary). Due to this, for both the gelatin itself and the systems formed by it, the variability of the structure and physicochemical properties are very characteristic. Fibrils formed by its molecules, in turn, form supramolecular assemblies that in vivo play the role of an extracellular matrix that functions as a kind of “supporting structure” and also participates in specific interactions with proteins, nucleic acids, and inorganic ions. The combination of both of these motifs makes gelatin a very convenient object for the implementation of various nanostructures and nanomaterials based on them. At the same time, this biopolymer is characterized by very easy biodegradability in natural conditions and the absence of toxicity, which is especially important in connection with the increasing burden on the environment from our civilization.

One of the most convenient options for creating nanosystems based on gelatin and/or its “progenitor”, collagen, is the construction of thin films using the Langmuir–Blodgett (LB-films) or Langmuir–Schaefer (LS-films) method. The obligatory first stage in the creation of such films is the preparation of monomolecular layers of a suitable composition and structure. When applied to the water–air interface, proteins form stable monomolecular layers, the properties of which are currently studied in detail [255,256,257,258,259,260,261,262,263,264,265,266,267,268,269,270]. In this connection, it should be noted that, in general, LB-films of proteins as potential functional biomaterials have attracted the close attention of researchers from as early as the end of the 20th century [271,272,273,274,275,276,277,278,279]. In particular, they were considered objects for recording and storing information, in micro-optics, microelectronics, and biotechnology [271,272,273], as highly sensitive biosensors [274,275,276]. In [277,278], the possibility of using highly ordered bacteriorhodopsin LB-films to create photosensors and record/store information was shown. However, advances in the creation of biosensors and bioelectronics significantly depend on the possibility of preparing protein layers of a given structure [279], so a very advanced technology for obtaining ordered LB-films of proteins in general and gelatin, in particular, should be developed to implement biomolecular electronic devices. However, the indisputable advantage of the monomolecular layer method is the ability to control the formation of ordered monolayers by controlling and measuring the two-dimensional pressure and area per molecule. It should be said that many doubts about the prospects of the method are easily removed if certain rules for the preparation and transfer of the monolayers of proteins, including both in general and gelatin in particular, are observed.

A very interesting point connected to the possibility of using gelatin in nanotechnology is the problem of realizing the liquid-crystal state in systems based on this biopolymer. As has long been known, the liquid-crystal state can, in principle, be realized for those substances whose molecules, on the one hand, have a sufficiently large length and small width and, on the other hand, when a very strong intermolecular interaction takes place between these molecules. By taking into account all that has been said in Section 2, there is no doubt that gelatin is one of these substances. The theory of the liquid-crystal state, developed for molecules in the form of rigid rod-shaped particles to which gelatin molecules belong, makes it possible to consider the formation of such a state for gelatin molecules or fibrils as quite acceptable [280,281,282]. A number of works carried out in the last century [283,284,285,286,287,288,289,290,291,292,293] were devoted to the study of liquid-crystalline structures of gelatin and its “progenitor”—collagen. The formation of such structures is facilitated by a decrease in water concentration, shear stresses, and magnetic and electric fields [294,295,296,297,298,299]. In conclusion to this section, we should note that interest in the use of gelatin as a basis for the construction of nanomaterials is not decreasing, as evidenced by the works published already in the current century, in particular [300,301,302,303,304,305,306,307,308,309,310,311,312,313,314,315,316,317,318,319,320,321,322,323,324,325]. In fairness, it should also be noted that there are certain difficulties associated with the use of gelatin as a basis for creating functional materials due to its relatively low mechanical strength. However, they may, in principle, be eliminated by combining gelatin with other polymers, “cross-linking” its molecules and/or modifying it with various fillers consisting of nanoparticles of various chemical compounds, among which are noteworthy, are such exotic compounds as carbon nanotubes, graphene, and graphene oxide [322].

## 8. Conclusions

As can be seen from all of the above, the totality of currently known information about gelatin gives every reason to call it the most famous substance among all proteins; and it was precisely this circumstance that became the main reason for this review article to be written. The availability of this substance, its popularity since very ancient times, and the unique physical, chemical, and biological properties of both itself and its numerous modified derivatives make it possible for use in various branches of science and practice, primarily in medicine, where it is the most an adequate vehicle for drug delivery and therapeutic applications. It should also be especially noted that using a variety of chemical reactions occurring in the gelatin matrix, it is possible to obtain a very large variety of gelatin-immobilized chemical compounds, which, as a rule, consist of nanoparticles. However, it has long been discovered that the reactivity of chemicals consisting of nanoparticles in chemical and biochemical processes (and, hence, the associated biological activity) is much higher compared to that of the same substances that consist of micro—or macroparticles. That is why gelatin-immobilized systems obtained by forming particles of a given substance directly in an array or a thin layer of a given biopolymer as a result of chemical transformations and having a nanoscale level of the organization seems to be more promising for use (primarily for targeted drug delivery to various tissues of the human body), rather than gelatin-immobilized systems obtained by the simple dispersion of particles or substances in an array or thin layer of gelatin (which, as a rule, have a micro-sized level of organization). The point is only to develop methods for obtaining drugs based on such systems and introduce them into wide medical practice.

As can be seen from all of the above, the significance of gelatin in modern anthropogenic activity in general and in the physico-chemistry of proteins, in particular, is very high; at the same time, it tends to further increase because the possibilities for the practical use of this remarkable substance are still far from being fully disclosed. In this review, of course, we were able to quote and at least briefly characterize by no means all publications devoted to gelatin, the total number of which at this point in time is difficult to determine even approximately (it is measured, at least a four-digit, and possibly even a five-digit number), and therefore the author of these lines considers it necessary to apologize to those researchers whose publications on this biopolymer were not mentioned in this review. This is partly due to the fact that when selecting the cited literature, we took into account whether a given source had a DOI index or another index that made it easy enough to find on the corresponding Internet website; for this reason, we had to exclude from consideration many such sources that, for one reason or another, did not have such an index at the time of writing this article and, accordingly, are not very accessible for familiarization with their content. However, be that as it may, gelatin has been and remains now one of the most important objects of study, located at the junction of three key fundamental scientific disciplines—chemistry, biology, and physics—and there is no doubt that in the future it will not only retain its importance in those areas of science and practices where it has already found an appropriate application but will also expand the list of these same areas.

## Data Availability

No unpublished data were created or analyzed in this article.
